# Elevated gamma-glutamyl transferase levels in early pregnancy increase pregnant women's risk of gestational hypertension and preeclampsia

**DOI:** 10.7189/jogh.16.04132

**Published:** 2026-04-24

**Authors:** Chuanlu Xu, Yuping Tang, Yirong Bao, Yuhong Li, Jia Zuo, Xiaohua Liu, Xiaoxian Qu, Hao Ying

**Affiliations:** 1 Department of Obstetrics, Shanghai Key Laboratory of Maternal Fetal Medicine, Shanghai Institute of Maternal-Fetal Medicine and Gynecologic Oncology, Shanghai First Maternity and Infant Hospital, School of Medicine, Tongji University, Shanghai, China.

## Abstract

**Background:**

The association between liver dysfunction in early pregnancy and the hypertensive disorders in pregnancy (HDP) remains unclear.

**Methods:**

This retrospective cohort study included all singleton pregnant women (n = 57 386) who underwent liver function tests (LFTs) at their first antenatal visit from six to 19^+6^ weeks of gestation. The exposure was liver dysfunction. The primary outcome was the development of hypertensive disorders of pregnancy (HDP), categorised into gestational hypertension and preeclampsia (PE). Preeclampsia was categorised into mild and severe forms, as well as early-onset and late-onset types. Multiple logistic regression analyses were conducted to estimate the risk for HDP and its subtypes across different liver function biomarker levels.

**Results:**

Among the 50 423 women included in the study, 17.1% had abnormal liver function. The overall HDP rate was 3.9%. Across the different serum Gamma-glutamyltransferase (GGT)groups, the HDP incidence rates were 2.9% (0–12 units per litre /U/L)), 5.4% (12.1–32 U/L), 10.0% (32.1–64 U/L), and 10.5% (>64 U/L) (*P* < 0.001 for trend). Compared with the reference range for GGT (0–12 U/L), the group with GGT levels of 12.1–32 U/L exhibited a 48.6% greater risk (adjusted odds ratio (OR) = 1.486; 95% confidence interval (CI) = 1.344–1.642), the group with GGT levels of 32.1–64 U/L had a 50.9% increased risk (aOR = 1.509; 95% CI = 1.375–1.657), and those with GGT levels >64 U/L had a 25.0% greater risk (aOR = 1.250; 95% CI = 1.078–1.449) of HDP. We further assessed the associations between varying GGT levels and HDP subtypes (gestational hypertension, mild PE, severe PE, early onset PE, and late-onset PE), and similar results were obtained for all subtypes. Restricted cubic spline (RCS) curves revealed that elevated serum GGT levels have a dose-response relationship with HDP, along with its subtypes.

**Conclusions:**

GGT levels during early pregnancy have a dose-response relationship with the development of HDP and its subtypes.

Despite extensive research efforts over the last decade, hypertensive disorders of pregnancy (HDP) have persisted as unpredictable complications affecting up to 10% of pregnant women globally, posing significant threats to maternal and perinatal health and accounting for substantial morbidity and mortality worldwide [1]. Effective clinical interventions beyond the vigilant monitoring of maternal symptoms for HDP management are limited [2]. Early identification of pregnant women at an increased risk of HDP could enable more intensive surveillance and timely interventions [3]. Consequently, investigation of modifiable risk factors is highly important for the early prevention of HDP.

Increasing awareness is emerging regarding the correlation between impaired liver function in early pregnancy and the onset of HDP [4–6]. The liver is a crucial metabolic organ in the human body and is involved in a multitude of processes, including the synthesis, breakdown, storage, and biotransformation of various biomolecules. Serum alanine aminotransferase (ALT), aspartate aminotransferase (AST), gamma-glutamyl transferase (GGT), total bilirubin (TBiL), and direct bilirubin (DBiL) are commonly employed as reliable indicators for assessing liver health [7] and are routinely screened for the evaluation of liver function during early pregnancy. Nevertheless, the correlations between these biomarkers during early pregnancy and the likelihood of developing HDP, along with its subtypes, are variable and not fully understood. A retrospective cohort study involving 2322 females from East Asia demonstrated that when the ALT level in early pregnancy exceeded the 95th percentile (30 U/L), the risk of preeclampsia (PE) tripled [4]. However, Liu et al. reported that the highest quartiles of GGT, ALP, and AST/ALT were significantly associated with a 1.71-fold (95% confidence interval (Cl) = 1.23–2.41), 1.53-fold (95% Cl = 1.10–2.14), and 0.62-fold (95% Cl = 0.43–0.90) increased risk of HDP, respectively, among 10 610 pregnant women. There was no significant association between the ALT or AST levels and the risk of HDP [5]. A prospective cohort study involving 1041 expectant mothers in South China indicated that elevated GGT and ALP levels during early pregnancy served as independent predictors for the development of gestational hypertension (GH) and PE [6]. Another prospective cohort study of 5685 pregnant women revealed that elevated levels of ALT, ALP, and GGT that still fall within the normal range during early pregnancy are associated with a heightened risk of GH and PE [8]. Studies on the association between these liver function biomarkers and the risk of HDP are inconsistent, and previous studies have not conducted further analyses of the different subtypes of PE.

In the current study, we explored the relationship between abnormal liver function test (LFT) results during early pregnancy and the risk of HDP based on a cohort study of Chinese women. HDP was subdivided into GH and PE, and PE was further systematically categorised into mild and severe PE, and early- and late-onset PE. The association between abnormal LFT results and risk of PE subtypes was further investigated.

## METHODS

### Study population

This cohort study included all pregnant women who had available medical records, regular check-ups, and delivered at Shanghai First Maternity and Infant Hospital, a tertiary care hospital affiliated with School of Medicine Tongji University in Shanghai, China between February 2013 to October 2017. The data was analysed on October 2023. Relevant information about the women, such as age, height, pre-pregnancy body weight, gestational week at the first antenatal visit, educational level, conception method, gravidity, and parity, was also recorded.

This cohort study included all singleton pregnant women who underwent LFTs at their first antenatal visit from 6 to 19^+6^ weeks of gestation (inclusive). The exclusion criteria were as follows:

(1) pre-existing chronic diseases, such as immunological disease, diabetes, or kidney ailments;

(2) infections with hepatotropic viruses, such as hepatitis B, C, and E viruses;

(3) chronic hypertension or blood pressure greater than 140/90 mm Hg at the first prenatal visit; and

(4) missing data on LFT results or pregnancy outcomes.

### Clinical and laboratory measurements

The classification of pre-pregnancy BMI was based on the adult body weight assessment document issued by the National Health and Family Planning Commission (its functions were integrated into the National Health Commission) of China as follows: underweight (<18.5 kg/m^2^), normal weight (18.5–23.9 kg/m^2^), overweight (24.0–27.9 kg/m^2^), and obese (28.0 kg/m^2^ or more) [9].

Liver function tests were performed at the hospital’s clinical laboratory using a HITACHI fully automatic biochemical analyser (008; Hitachi, Tokyo, Japan). The reference values for liver function biomarkers were as follows: ALT = 1–40 U/L; AST = 2–40 U/L; GGT = 0–32 U/L; TBiL = 0–17.1 μmol/L; and DBiL = 0–6.0 μmol/L. An abnormal LFT result was defined as a value of at least one of the five markers (ALT, AST, GGT, TBiL and DBiL) being larger than the upper limit of normal (ULN), *i.e*. an ALT level >40 U/L, an AST level >40 U/L, a GGT level >32 U/L, a TBiL level >17.1 μmol/L, or a DBiL level >6.0 μmol/L.

### Statistical analysis

The diagnosis of GH and PE was made based on the criteria set by the American College of Obstetricians and Gynecologists (ACOG) [10]. Preeclampsia was subdivided into mild and severe PE based on signs and symptoms and into early onset (onset at <34 weeks of gestation) and late-onset PE (onset at ≥34 weeks of gestation) [10].

Continuous data were presented as mean ± standard deviation (SD) (normal distribution) or median (min–max) (non-normal), and categorical data as n (%). Normality was assessed using the Shapiro-Wilk test and histograms/Q-Q plots. Group comparisons between women with and without HDP were conducted using Student’s *t* test (normal continuous), Mann-Whitney U test (non-normal continuous), and χ^2^ or Fisher exact test (categorical), as appropriate. For multi-level categorical variables, significant overall χ^2^ results (*P* < 0.05) were followed by post-hoc pairwise comparisons using adjusted residuals; an absolute adjusted residual >1.96 indicated statistical significance (α = 0.05).

Univariate analysis (Model I) and multiple logistic regression analysis (Model II) were used to estimate the unadjusted and adjusted odds ratios (ORs) and 95% confidence intervals (CIs) of the HDP incidence rate for different exposure groups with different biomarker levels. In accordance with the ACOG Clinical Guidelines [11], ALT and AST levels were categorised into various degrees of elevation. Borderline elevation of both ALT and AST levels was defined as 41–80 U/L (approximately 2 × ULN), mild elevation as 81–200 U/L, and moderate or severe elevation as >200 U/L. This categorisation aided in assessing the severity of liver function abnormalities by referencing the enzyme levels. The association between GGT level and HDP incidence was assessed using the following categories: 0–12 U/L (representing the median GGT level), 12.1–32 U/L (1 × ULN), 32.1–64 U/L (2 × ULN), and >64 U/L. Multiple logistic regression analyses (Model II) were conducted for various GGT levels to account for the potential risk factors across all HDP categories. Statistical analyses were performed using IBM SPSS Statistics for Windows, version 23.0 (IBM Corp., Armonk, NY, USA).

Additionally, we explored the relationship between maternal serum GGT levels and the probabilities of all HDP categories using restricted cubic spline (RCS) curves. These analyses enabled us to produce OR curves to comprehensively depict relationships across the entire range. Statistical computations were performed using the *R*, version 3.6.1. (R Foundation for Statistical Computing, Vienna, Austria).

## RESULTS

**Figure 1:**
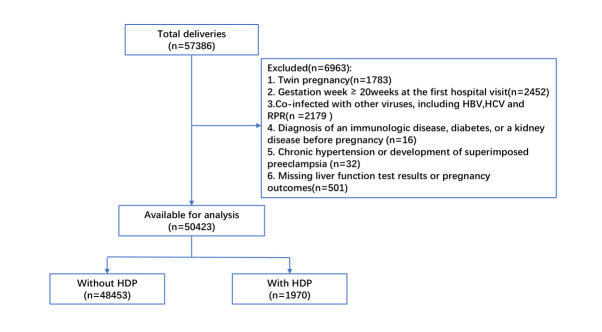
Flowchart of study participants.

Among the 57 386 singleton pregnant women who first visited our institution and underwent preliminary laboratory tests during early pregnancy, 6963 were excluded because of various factors. Overall, 50 423 women (96.1%) were included in the final analysis. Within this cohort, 1970 (3.9%) women developed HDP during pregnancy (**Figure 1**).

There was no significant difference in the gestational week of the first antenatal care visit between the women with and without HDP. However, the women in the HDP group were older, heavier, less educated, less gravid, parous, and had greater assisted reproductive technology use. Additionally, women in the HDP group had higher systolic and diastolic pressures at their first antenatal care visits (**Table 1**).

**Table 1:** Baseline characteristics of the study participants with and without HDP

Variables at the first antenatal care visit	Without HDP	With HDP	*P*-value
N (%)	48 453 (96.1)	1970 (3.9)	
Gestational weekt, weeks, mean ± SD	15.0 ± 1.5	15.0 ± 1.5	0.892*
Age, years, mean ± SD	30.3 ± 3.7	30.6 ± 3.8	<0.001*
Age group, years, n (%)			0.006†
*<18*	3 (0.006)	0 (0)	
*18–35*	41 965 (86.6)	1657 (84.1)	
*35–40*	5843 (12.1)	275 (14.0)	
*≥40*	640 (1.3)	38 (1.9)	
*Missing*	2 (0.004)	0 (0)	
Prepregnancy BMI, kg/m^2^, mean ± SD	21.8 ± 2.8	23.9 ± 3.8	<0.001*
BMI group, kg/m^2^, n (%)			<0.001†
*Underweight (<18.5)*	4209 (8.7)	79 (4.0)	
*Normal weight (18.5–23.9)*	34 847 (71.9)	1021 (51.8)	
*Overweight (24.0–27.9)*	7705 (15.9)	590 (29.9)	
*Obese (≥28)*	1434 (3.0)	265 (13.5)	
*Missing*	258 (0.5)	15 (0.8)	
Education level, n (%)			0.002†
*High school/under*	3030 (6.3)	161 (8.2)	
*College/above*	40 514 (83.6)	1606 (81.5)	
*Missing*	4909 (10.1)	203 (10.3)	
Assisted reproductive technology use, n (%)			<0.001†
*Yes*	1412 (2.9)	120 (6.1)	
*No*	47 041 (97.1)	1850 (93.9)	
Gravidity, n (%)			0.008†
*1*	26 531 (54.8)	1156 (58.7)	
*2*	12 884 (26.6)	480 (24.4)	
*3*	5714 (11.8)	208 (10.6)	
*≥4*	3323 (6.9)	126 (6.4)	
Parity, n (%)			<0.001†
*1*	37 810 (78.0)	1721 (87.4)	
*2*	10 429 (21.5)	241 (12.2)	
*≥3*	214 (0.4)	8 (0.4)	
Blood pressure at the first antenatal care visit, mean ± SD			
*Systolic pressure*	104.49 ± 10.52	109.48 ± 12.0	<0.001*
*Diastolic pressure*	67.8 ± 10.00	71.47 ± 8.22	<0.001*
Liver function biomarkers at the first antenatal care visit, median (min–max)			
*ALT (U/L)*	16 (1.0–592)	19 (2.0–342)	
*AST (U/L)*	18 (3.0–265)	18 (8.0–132)	
*GGT(U/L)*	12 (1.0–279)	15 (4–150)	
*TBiL (μmol/L)*	7.4 (0.3–61.4)	6.9 (2.0–28.2)	
*DBiL (μmol/L)*	2.0 (0–30.8)	1.9 (0.4–7.9)	

ALT – alanine aminotransferase, AST – aspartate aminotransferase, BMI – body mass index, DBiL – direct bilirubin, GGT – gamma-glutamyltransferase, TBiL – total bilirubin

*Derived from Student’s t- test.

†Derived from the χ^2^ test or Fisher Exact Test.

Significant variations of HDP incidence were observed for both ALT (*P* = 0.001) and GGT (*P* < 0.001). Notably, HDP incidence demonstrated a progressive increase with higher GGT categories, rising from 2.7% (≤12 U/L) to 10.5% (>64 U/L). No significant associations were found for AST, TBiL, or DBiL (all *P* > 0.05) (Table S1 in the **Online Supplementary Document**).

Upon analysing the HDP subcategories (GH, mild PE, and severe PE), notable variations in the rates of GH and mild PE across the different ALT groups emerged. Specifically, the *P* for trend was 0.032 for GH and *P* for trend  was 0.001 for mild PE. However, no significant differences were found in severe PE rates with a *P* for trend,  of  0.912. Significant differences were observed among the various GGT groups for all HDP subcategories (trend, *P* < 0.001 for GH, mild PE, and severe PE). No significant differences were detected among the AST, TBiL, and DBiL groups for any of the HDP subcategories (Table S1 in the **Online Supplementary Document**).

We further analysed the PE subcategories (early onset PE and late-onset PE) based on the week of onset and detected significant differences in late-onset PE among the different ALT groups (trend *P* = 0.015); however, no significant differences were detected for early onset PE (trend *P* = 0.203). Significant differences were observed between the different GGT groups (*P* < 0.001 for early- and late-onset PE). No significant differences were detected between the AST, TBiL, and DBiL groups (Table S1 in the **Online Supplementary Document**).

Post-hoc pairwise comparisons for ALT and GGT categories were made. For ALT, compared to the reference group (0–40 U/L), the 40–80 U/L group showed a significantly higher occurrence of mild preeclampsia (adjusted residual = +3.0, *P* < 0.05) and late-onset severe preeclampsia (adjusted residual = +2.7, *P* = 0.007). No significant associations were observed for early-onset severe preeclampsia (Table S2–3 in the **Online Supplementary Document**). For GGT, compared to the lowest group (0–12 U/L), all elevated GGT categories (12.1–32 U/L, 32.1–64 U/L, and >64 U/L) demonstrated significantly higher occurrences of all HDP subtypes, including gestational hypertension, mild preeclampsia, and severe preeclampsia (all adjusted residuals>|2.6|, *P* < 0.01). The strongest associations were observed in the 32.1–64 U/L group. Regarding severe PE subtypes, women with GGT≥32.1 U/L had significantly higher occurrences of both early- and late-onset severe PE, whereas the 12.1–32 U/L group was associated only with late-onset severe PE (Table S4–5 in the **Online Supplementary Document**).

Univariate analysis was performed to estimate the ORs and 95% CIs for HDP across various exposure groups based on the ALT, AST, GGT, TBiL, and DBiL levels. Compared to ALT levels within the reference range (0–40 U/L), only ALT levels between 41 and 80 U/L were associated with a 28.5% increased risk of HDP (OR = 1.285; 95% CI = 1.119–1.475). However, after adjusting for confounding factors, no significant increase in HDP risk was found for ALT levels between 41 and 80 U/L (aOR = 1.053; 95% CI = 0.914–1.212) (**Table 2**).

**Table 2:** Unadjusted and adjusted associations between liver function biomarkers and the risk of HDP

LFT	HDP N (%)	Model 1 OR (95% CI) *	Model 2, aOR (95% CI) †
ALT (U/L) (n = 50 423)			
*0–40*	1648 (3.8)	1.0 ref	1.0 ref
*41–80*	243 (4.8)	1.285 (1.119–1.475)	1.053 (0.914–1.212)
*81–200*	76 (4.7)	1.115 (0.991–1.254)	1.059 (0.834–1.345)
*>200*	3 (3.4)	0.965 (0.657–1.417)	0.677 (0.212–2.164)
P for trend		0.001	0.545
AST (U/L) (n = 50 423)			
*0–40*	1852 (3.9)	1.0 ref	1.0 ref
*41–80*	108 (3.9)	1.179 (0.990–1.404)	0.909 (0.744–1.110)
*81–200*	10 (3.1)	1.182 (0.942–1.484)	0.845 (0.614–1.161)
*>200*	0 (0)	-	-
P for trend		0.625	0.221
GGT (U/L) (n = 50 342)			
*0.0–12.0*	744 (2.7)	1.0 ref	1.0 ref
*12.1–32.0*	1012 (4.9)	1.888 (1.715–2.079)	1.486 (1.344–1.642)
*32.1–64.0*	187 (10.0)	2.008 (1.847–2.184)	1.509 (1.375–1.657)
*>64.0*	25 (10.5)	1.621 (1.409–1.865)	1.250 (1.078–1.449)
P for trend		<0.0001	<0.0001
TBiL (μmol/L) (n = 50 419)			
*0.0–17.1*	1949 (3.9)	1.0 ref	1.0 ref
*>17.2*	21(3.9)	0.997(0.643-1.546)	1.149(0.730-1.808)
DBiL (μmol/L) (n = 50 419)			
*0.0–6.0*	1965(3.9)	1.0 ref	1.0 ref
*>6.0*	5(2.3)	0.576(0.237-1.400)	1.642(0.263-1.570)

ALT – alanine aminotransferase, aOR – adjusted odds ratio, AST – aspartate aminotransferase, CI – confidence interval, DBiL – direct bilirubin, GGT – gamma-glutamyltransferase, HDP – hypertensive disorders of pregnancy, LFT – liver function test, OR – odds ratio, TBiL – total bilirubin

*An unadjusted model.

†Adjusted for confounding variables including age, prepregnancy body mass index, education level, assisted reproductive technology use, gravidity, and parity.

Participants were categorised into groups with GGT levels of 0–12, 12.1–32, 32.1–64 U/L, and >64 U/L. With pregnant women with GGT levels of 0–12 U/L as the reference, pregnant women with GGT levels ranging from 12.1–32 U/L presented an 88.8% increased risk of HDP (OR = 1.888; 95% CI = 1.715– 2.079). Similarly, pregnant women with GGT levels of 32.1–64 U/L presented a 2-fold increased risk of HDP (OR = 2.008; 95% CI = 1.847–2.184), and the group with GGT levels >64 U/L presented a 62.1% increased risk (OR = 1.621; 95% CI = 1.409– 1.865) (**Table 2**).

After adjustment for confounders, compared with the group with GGT levels of 0–12 U/L as the reference, the group with GGT levels of 12.1–32 U/L exhibited a 48.6% greater risk (aOR = 1.486; 95% CI = 1.344–1.642), the group with GGT levels of 32.1–64 U/L showed a 50.9% increased risk (aOR = 1.509; 95% CI = 1.375–1.657), and those with GGT levels >64 U/L had a 25.0% greater risk of HDP (aOR = 1.250; 95% CI = 1.078–1.449). Conversely, no significant association was found between the risk of HDP and varying AST, TBiL, or DBiL levels (**Table 2**).

We further assessed the association between GGT levels and the HDP subtype. In the group with GGT levels of 0–12 U/L as the reference, elevated GGT levels (12.1–32 U/L) were associated with a 45.8% increased risk of GH (aOR = 1.458; 95% CI = 1.270–1.674), 60.0% increased risk of mild PE (aOR = 1.600; 95% CI = 1.306–1.960), 43.7% increased risk of severe PE (aOR = 1.437; 95% CI = 1.181–1.749), 52.3% increased risk of early onset PE (aOR = 1.523; 95% CI = 0.913–2.540), and 51.4% increased risk of late-onset PE (aOR = 1.514; 95% CI = 1.306–1.754) (*P* < 0.001). For elevated GGT levels (32.1–64 U/L) and GGT levels >64 U/L, similar results were obtained for all subtypes (**Table 3**).

**Table 3:** Adjusted associations between GGT levels and the risk of HDP subtypes*

GGT (U/L)	GH, aOR (95% CI)	Mild PE, aOR (95% CI)	Severe PE, aOR (95% CI)	Early-onset PE, aOR (95% CI)	Late-onset PE, aOR (95% CI)
0.0–12.0	1.0 ref	1.0 ref	1.0 ref	1.0 ref	1.0 ref
12.1–32.0	1.458 (1.270–1.674)	1.600 (1.306–1.960)	1.437 (1.181–1.749)	1.523 (0.913–2.540)	1.514 (1.306–1.754)
32.1–64.0	1.469 (1.297–1.664)	1.700 (1.423–2.032)	1.389 (1.136–1.698)	1.811 (1.186–2.766)	1.528 (1.328–1.759)
>64.0	1.143 (0.929–1.405)	1.411 (1.079–1.845)	1.342 (1.010–1.783)	1.975 (1.281–3.043)	1.297 (1.038–1.621)
p for trend	<0.001	<0.001	<0.001	<0.001	<0.001

CI – confidence interval, GGT – gamma-glutamyltransferase, GH – gestational hypertension, OR – odds ratio, PE – preeclampsia

*The models were adjusted for confounding variables including age, prepregnancy body mass index, education level, assisted reproductive technology use, gravidity, and parity.

**Figure 2:**
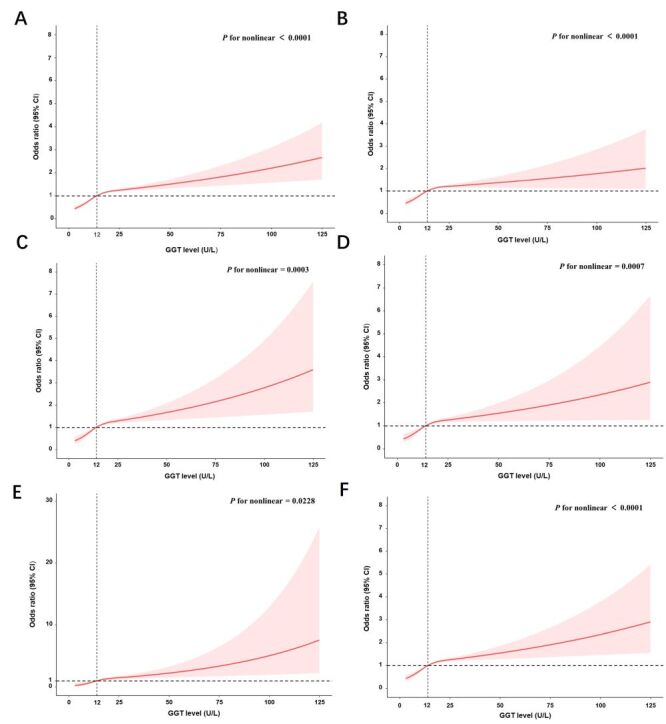
Restricted cubic spline curves with logistic regression of the dose-response relationship between maternal serum GGT levels and the risk of (**Panel A**) HDP, (**Panel B**) GH, (**Panel C**) mild PE, (**Panel D**) severe PE, (**Panel E**) early-onset PE, and (**Panel F**) late-onset PE.The models were adjusted for maternal age, prepregnancy BMI, education level, use of assisted reproductive technology, gravidity, and parity. CI – confidence interval, GGT – gamma-glutamyltransferase, GH gestational hypertension, HDP – hypertensive disorders of pregnancy, OR – odds ratio, PE – preeclampsia.

We investigated the dose-response relationship between maternal serum GGT levels and the likelihood of all HDP subtypes by employing RCS curves, with a median GGT level of 12.0 U/L used as the reference. Notably, statistically significant nonlinear effects were identified for all HDP subtypes, with the following *P* values indicating nonlinearity: HDP (*P* < 0.001) (**Figure 2**, Panel A), GH (*P* < 0.001) (**Figure 2**, Panel B), mild PE (*P* < 0.001) (**Figure 2**, Panel C), severe PE (*P* < 0.001) (**Figure 2**, Panel D), early onset PE (*P* = 0.023) (**Figure 2**, Panel E), and late-onset PE (*P* < 0.001) (**Figure 2**, Panel F). The models were adjusted to account for factors such as maternal age, prepregnancy body mass index, education level, use of assisted reproductive technology, gravidity, and parity (**Figure 2**).

## DISCUSSION

This study revealed a positive association between abnormal LFT results in early pregnancy and subsequent risk of HDP in a cohort of pregnant Chinese women. The results revealed that elevated serum GGT levels were associated with subsequent development of HDP. Similarly, our study uncovered a dose-dependent association between increased GGT levels and the occurrence of HDP, along with all subtypes. Few studies have investigated the relationship between abnormal LFT results and incidence of HDP subtypes. Our study contributes to the literature by exploring the relationship between abnormal LFT results during early pregnancy and pregnancy complications. This study provides novel evidence to enhance our understanding of the association between abnormal LFT results in early pregnancy and subsequent HDP development. Our results support the use of maternal GGT measurements in early pregnancy serve as a simple, low-cost indicator for heightened surveillance to stratify the risk of HDP, enabling targeted antenatal surveillance in high-risk groups and early detection and preventive measures. In addition, these findings suggest that the positive correlations between GGT levels in early pregnancy and the incidence of HDP subtypes persist even within the normal range of liver function. At present, clinical reference values for GGT are established based on a broad population group encompassing individuals of both sexes; however, liver enzyme levels physiologically decrease during pregnancy [12], prompting questions regarding the applicability and validity of these references in women in early pregnancy.

The rate of abnormal LFT results in our study was 17.1%, which aligns with the results obtained in previous studies. A large proportion of asymptomatic individuals with abnormal LFT results during early pregnancy remain undiagnosed [13]. The most common aetiology is metabolic dysfunction-associated steatotic liver disease (MASLD), with a reported occurrence rate of 16–18% in pregnant women, followed by drug-induced liver injury, autoimmune hepatitis, infection with hepatotropic virus, and other liver diseases [14]. Although liver biopsy is the definitive method for evaluating MASLD, testing ALT, AST, and GGT levels seems to be a reasonable non-invasive surrogate measure in epidemiological studies [15]. The biological mechanisms by which abnormal LFT results increase HDP risk are poorly understood, and our findings provide insights into this topic [16]. Although the initial clinical manifestations of HDP are observed during pregnancy, especially after 20 weeks of gestation, this condition originates mainly during early pregnancy [17]. According to previous studies, local and systemic inflammation during early pregnancy may be linked to later placental occurrence, and significant systemic abnormalities in mothers may play a role in the onset of HDP.

The role of liver function biomarkers as risk factors for HDP is not well understood. Elevated levels of one or more of these biomarkers (ALT, AST, TBiL, and DBiL) suggest hepatocellular injury and/or biliary obstruction, which require assessment and potential evaluation. Recent studies of elevated ALT or AST levels and HDP risk have produced varying and inconsistent results. In the present study, which included a large sample size, we did not find that abnormal serum ALT, AST, TBiL, or DBiL levels increased HDP risk. This discrepancy might be due to differences in study design, diagnostic criteria, sample size, or population characteristics (maternal age, ethnicity, gestational week, and lifestyle habits) [6]. Increasing evidence from animal research indicates that bilirubin possesses anti-complement properties and is capable of inhibiting cellular inflammatory responses [18,19]. Inflammation plays a crucial role as a mediator of HDP onset and contributes to its underlying pathological processes. An increase in serum levels of TBiL or DBiL may prevent the progression of HDP owing to their anti-inflammatory effects. Further high-quality studies with larger sample sizes are needed to validate the relationships between these biomarkers (ALT, AST, TBiL, and DBiL) and HDP.

GGT serves as an indicator of latent liver conditions (*e.g*. MASLD), metabolic syndrome, and increased oxidative stress [20]. Apart from the liver, GGT is also present and expressed in various other tissues such as the placenta, lungs, and pancreas [21]. As a key enzyme in glutathione metabolism, GGT facilitates the extracellular degradation of this thiol antioxidant, thereby preventing ROS-induced damage to critical cellular structures [22,23]. Our study revealed dose-response relationships between elevated GGT levels in early pregnancy and all HDP subtypes. When the subtypes of PE were compared, early onset PE was more closely related to elevated GGT levels than was late-onset PE. Similarly, mild PE was more closely associated with increased GGT levels than was severe PE. Nevertheless, the exact mechanisms that explain the connection between increased GGT levels and varying symptom severity, as well as the gestational week of HDP onset, remain unknown. Elevated GGT levels may lead to endothelial damage and vascular sclerosis via enhanced oxidative stress in the placenta and peripheral blood vessels, thereby posing a significant risk for PE [24–26]. Additional research is required to validate the hypothesised mechanisms linking elevated GGT levels during early pregnancy to the occurrence of HDP in a broader population. In summary, these findings highlight the crucial need to increase awareness of the correlation between elevated GGT levels during early pregnancy and the development of HDP.

The main pathological feature of HDP is inadequate trophoblastic infiltration of the uterus, leading to subsequent alterations in uteroplacental blood flow. This leads to dysfunction of the maternal endothelium and initiates the manifestation of the clinical symptoms of the disease [27]. Currently, there is a lack of effective predictors of the development of HDP in pregnant women during early pregnancy. Background characteristics (*e.g*. obesity, immunological disease, diabetes, or kidney disease) or past history (*e.g*. a history of PE or a family history of PE) were used to identify pregnant women at a high risk of HDP. To enhance the precision of prediction, it is necessary to employ a blend of innovative serum markers originating from the placenta, including levels of serum pregnancy-associated plasma protein-A (PAPP-A) and placental growth factor (PlGF), along with screening outcomes from Doppler ultrasound examination of the uterine artery [28]. However, considerable time is required for these tests to become widely accessible. Using easily measurable GGT levels as a potential predictor of HDP and encouraging the use of low-dose aspirin could serve as a viable approach to prevent the development of HDP. Additional studies are required to assess the significance of GGT level in predicting HDP and the preventive effects of aspirin. In addition, GGT is a well-established serum marker for MASLD and metabolic syndrome, and certain factors originating from adipose tissue might influence systemic inflammation and lead to HDP [29]. Novel approaches such as statin treatment, which have already undergone trials [30], may also be an effective strategy to prevent the development of HDP [31].

Our study has two significant strengths. First, we examined the connections between abnormal liver function biomarkers in early pregnancy and systematic stratification of HDP into clinically relevant subtypes (GH, mild/severe PE, early/late-onset PE) allows for a more nuanced understanding of the associations, improving the clinical applicability of our findings. Second, the use of a large, homogeneous cohort with standardised data collection from a tertiary centre; the comprehensive adjustment for a wide array of potential confounders (*e.g*. maternal age, prepregnancy BMI, parity, etc) in our multivariate analyses strengthens the robustness of the observed associations. However, this study also had some limitations. First, the aetiology of abnormal LFTs in early pregnancy remains speculative. Although viral hepatitis has been ruled out, the proportion of other potential causes, such as autoimmune hepatitis, alcohol-related liver disease, and drug-induced liver injury, remains unclear. Second, we seldom obtained all the pertinent and desired variables. For instance, variables such as insulin resistance, lipid profiles, steatosis, smoking, alcohol, diet, physical activity, socioeconomic status, which could have been included as covariates, were not available. GGT likely serves as an indicator of underlying metabolic dysfunction rather than an independent risk factor; future studies should therefore account for these covariates to more clearly define the role of GGT. Third, the single-centre, retrospective design may limit the generalisability (external validity) of our findings to other populations with different demographics or health care settings. The findings should be interpreted with caution, and additional investigations involving diverse racial and ethnic populations are warranted to validate the generalisability of the findings of this study.

## CONCLUSIONS

In conclusion, among the liver function biomarkers of liver injury, serum GGT levels during early pregnancy have a - dose-response relationship with the incidence of HDP and all its subtypes in Chinese adults. Therefore, monitoring GGT levels during early pregnancy may be helpful for the early forecasting and prevention of HDP.

## Additional material


Online Supplementary Document
