# Supplementary material for: Elevated gamma-glutamyl transferase levels in early pregnancy increase pregnant women's risk of gestational hypertension and preeclampsia
Source: J Glob Health. 2026 Apr 24;16:04132. doi: 10.7189/jogh.16.04132 (PMC13107083; doi:10.7189/jogh.16.04132)
Supplement: Online Supplementary Document [file jogh-16-04132-s001.pdf]

Supplement to: Xu C, Tang Y, Bao Y, Li Y, Zuo J, Liu X, Qu X, Ying H. Elevated gamma-glutamyl transferase levels in early pregnancy increase pregnant women's risk of gestational hypertension and preeclampsia. J Glob Health. 2026;16:04132.

Table S1. Incidence of HDP and its subtypes across categories of liver function biomarkers

LFT	Total	HDP	GH	Mild PE	Severe PE	Early-onset PE	Late-onset PE
N (%)	50423 (100%)	1970 (3.9%)	1028 (2.0%)	474 (0.9%)	468 (0.9%)	76 (0.2%)	866 (1.7%)
ALT (U/L) (n=50423)							
0–40	43638 (86.6%)	1648 (3.8%)	865 (2.0%)	381 (0.9%)	402 (0.9%)	61 (0.1%)	722 (1.7%)
41–80	5063 (10.0%)	243 (4.8%)	121 (2.4%)	67 (2.4%)	55 (1.1%)	12 (0.2%)	110 (2.2%)
81–200	1634 (3.2%)	76 (4.7%)	41 (2.5%)	25 (1.5%)	10 (0.6%)	3 (0.2%)	32 (2.0%)
>200	88 (0.2%)	3 (3.4%)	1 (1.1%)	1 (1.1%)	1 (1.1%)	0 (0%)	2 (2.3%)
p for trend	-	0.001	0.032	<0.001	0.912	0.203	0.015
AST (U/L) (n=50423)							
0–40	47329 (93.9%)	1852 (3.9%)	967 (2.0%)	441 (1.0%)	444 (0.9%)	67 (0.1%)	818 (1.7%)
41–80	2766 (5.5%)	108 (3.9%)	56 (2.0%)	29 (1.0%)	23 (0.8%)	9 (0.3%)	43 (1.6%)
81–200	323 (0.6%)	10 (3.1%)	5 (1.5%)	4 (1.2%)	1 (0.3%)	0 (0%)	5 (1.5%)
>200	5 (0.01%)	0 (0%)	0 (0%)	0 (0%)	0 (0%)	0 (0%)	0 (0%)
p for trend	-	0.625	0.657	0.437	0.254	0.114	0.469
GGT (U/L) (n=50342)							
0-12.0	27760 (55.1%)	744 (2.7%)	385 (1.4%)	167 (0.7%)	192 (0.6%)	27 (0.1%)	332 (1.2%)
12.1-32	20473 (40.7%)	1012 (4.9%)	525 (2.6%)	250 (1.2%)	237 (1.2%)	37 (0.2%)	450 (2.2%)
32.1-64	1871 (3.7%)	187 (10.0%)	104 (5.6%)	50 (2.7%)	33 (1.8%)	9 (0.5%)	74 (4.0%)
>64	238 (0.5%)	25 (10.5%)	12 (5.0%)	7 (2.9%)	6 (2.5%)	3 (1.3%)	10 (4.2%)
p for trend	-	<0.001	<0.001	<0.001	<0.001	<0.001	<0.001
TBiL (μmol/L) (n=50419)							
0-17.1	49880 (98.9%)	1949 (3.9%)	1015 (2.0%)	471 (1.0%)	463 (0.9%)	76 (0.2%)	858 (1.7%)

>17.1	539 (1.1%)	21 (3.9%)	13 (2.4%)	3 (0.6%)	5 (0.9%)	0 (0%)	8 (1.5%)
p value	-	0.989	0.547	0.357	0.999	0.365	0.680
DBiL (μmol/L) (n=50419)							
0-6.0	50201 (99.6%)	1965 (3.9%)	1026 (2.0%)	474 (0.9%)	465 (0.9%)	76 (0.2%)	863 (1.7%)
>6.0	218 (0.4%)	5 (2.3%)	2 (0.9%)	0 (0%)	3 (1.4%)	0 (0%)	3 (1.4%)
p value	-	0.218	0.237	0.148	0.513	0.562	0.681

Abbreviation: ALT–alanine aminotransferase; AST–aspartate aminotransferase; DBiL–direct bilirubin; GGT–gamma-glutamyl-transferase; GH–gestational hypertension; HDP–hypertension disorders of pregnancy; LFT–liver function test; PE–preeclampsia; TBiL– total bilirubin.
- Not applicable; the "Total" column represents the overall cohort distribution.

Table S2. Post-hoc pairwise comparisons of ALT categories and HDP subtypes based on adjusted residuals

**ALT (U/L)**	**HDP Subtype**	**Adjusted Residual**	**p-value***
0–40	Mild PE	–4.0	<0.001
0–40	Gestational Hypertension	–2.3	0.021
40–80	Mild PE	+3.0	0.003
80–200	Mild PE	+2.5	0.012
40–80	Gestational Hypertension	+1.9	0.057

Note: Adjusted residuals > |1.96| correspond to $p < 0.05$. Only significant or marginal comparisons are shown. PE: Preeclampsia.

Supplementary Table S3. Post-hoc pairwise comparisons of ALT categories and severe preeclampsia subtypes based on adjusted residuals

**ALT (U/L)**	**Preeclampsia Subtype**	**Adjusted Residual**	**p-value***
0–40	Late-onset severe PE	–2.8	0.005

ALT (U/L)	Preeclampsia Subtype	Adjusted Residual	p-value*
40–80	Late-onset severe PE	+2.7	0.007
0–40	Early-onset severe PE	–1.6	0.110
40–80	Early-onset severe PE	+1.7	0.089
80–200	Late-onset severe PE	+0.8	0.424
>200	Late-onset severe PE	+0.4	0.690

Note: Adjusted residuals > |1.96| correspond to p < 0.05. Only significant comparisons are interpreted. PE: Preeclampsia.

Supplementary Table S4: Post-hoc Pairwise Comparisons for GGT Categories and HDP Subtypes

GGT (U/L)	HDP Subtype	Adjusted Residual	p-value*
0–12	Gestational Hypertension	–11.5	<0.001
0–12	Mild Preeclampsia	–8.8	<0.001
0–12	Severe Preeclampsia	–6.2	<0.001
12.1–32	Gestational Hypertension	+6.9	<0.001
12.1–32	Mild Preeclampsia	+5.4	<0.001
12.1–32	Severe Preeclampsia	+4.4	<0.001
32.1–64	Gestational Hypertension	+11.0	<0.001
32.1–64	Mild Preeclampsia	+7.9	<0.001
32.1–64	Severe Preeclampsia	+3.8	<0.001
>64	Gestational Hypertension	+3.3	0.001
>64	Mild Preeclampsia	+3.2	0.001

GGT (U/L)	HDP Subtype	Adjusted Residual	p-value*
>64	Severe Preeclampsia	+2.6	0.009

Note: Adjusted residuals > |1.96| correspond to $p < 0.05$. All comparisons shown are statistically significant. The reference group for comparison is the "normal" outcome category within each HDP subtype.

Supplementary Table S5: Post-hoc Pairwise Comparisons for GGT Categories and Severe Preeclampsia Subtypes

GGT (U/L)	Severe Preeclampsia Subtype	Adjusted Residual	p-value*
0–12	Early-onset severe PE	–3.5	<0.001
0–12	Late-onset severe PE	–10.3	<0.001
12.1–32	Early-onset severe PE	+1.5	0.134
12.1–32	Late-onset severe PE	+7.0	<0.001
32.1–64	Early-onset severe PE	+3.9	<0.001
32.1–64	Late-onset severe PE	+7.9	<0.001
>64	Early-onset severe PE	+4.5	<0.001
>64	Late-onset severe PE	+3.1	0.002

Note: Adjusted residuals > |1.96| correspond to $p < 0.05$. PE = preeclampsia.
